# Intergenerational transmission of parental child-rearing gender-role attitudes and its influence on gender roles in single-parent families

**DOI:** 10.1186/s40359-024-01594-z

**Published:** 2024-02-26

**Authors:** I-Jun Chen, Xiaoxiao Wang, Zhiyin Sun, Panlin Tang, Peiyi Chen

**Affiliations:** School of Education, Soochow University, Suzhou Industrial Park (SIP), Dushuhu Campus, No.1, Wenjing Road, 215123 Suzhou City, Jiangsu Province China

**Keywords:** Single-parent family, Parental child-rearing gender-role attitude, Intergenerational transmission, Gender role

## Abstract

**Background:**

The development of children’s gender roles in single-parent families is worthy of attention. It may be affected by family members’ gender roles and parental child-rearing gender-role attitudes (PCGA). PCGA will form a consistent or inconsistent intergenerational relationship between parents and children.

**Objective:**

This study examined the intergenerational similarities in gender roles and PCGA. Also, the intergenerational transmission of parental child-rearing gender-role attitudes (ITPCGA) in single-parent families, and the impact of various family factors on children’s gender roles were comprehensively considered.

**Method:**

Participants were 550 single-parent parent-adolescent dyads. The Gender-role Scale and the Parental Child-rearing Gender-role Attitude Scale were used to evaluate participants’ gender-role and PCGA. Chi-square tests and logistic regression analyses were used to examine the intergenerational similarities in gender roles and PCGA, and the influencing family factors of ITPCGA and children’s gender roles.

**Results:**

The intergenerational similarities of gender role types and PCGA types existed. Both parents’ gender roles and family gender pairs affected ITPCGA, father-daughter families and parents’ undifferentiated and sex-typed gender roles significantly predicted undesirable ITPCGA. Family gender pair, parent’s gender roles and ITPCGA types affected children’s gender roles. Undesirable ITPCGA significantly predicted children’s undifferentiated gender roles; father-daughter families and mother-son families, parents’ undifferentiated and sex-typed gender roles significantly predicted children’s sex-typed gender roles, and mother-son families and parents’ reversed gender roles significantly predicted children’s reversed gender role.

**Conclusions:**

This study highlights the effects of single-parent family gender pairs and parents’ gender roles on ITPCGA, which influences the development of children’s gender roles.

## Introduction

With the rapid development of the economy and society, the concept of marriage has changed significantly, divorce in China has become increasingly common [1]. China’s divorce rate has been rising for 16 consecutive years. According to data from 2020, there were 4.339 million couples who filed for divorce, and the crude divorce rate reached 3.1‰ [2]. The rising number of divorced couples reflects the increasing number of single-parent families [3]. Current research on single-parent families has focused on its effects on single-parent children, such as on their personality, their physiological and psychological development [4], internalization problem behavior [5], academic achievement [6], the development of social adaptation [7] and gender socialization process [8]. Gender roles, as a set of behavioral norms corresponding to one’s own gender, acquired through imitation and learning in the process of socialization, are an important part of the development of individual gender socialization [8, 9]. Therefore, in the context of an absent parent within single-parent families, gender roles are an issue that cannot be ignored when exploring the individual development.

The Structural-Functionalism viewed the family is an example of a subsystem that functions, or operates, for the survival and maintenance of society [10]. Structure is the arrangement of the roles of which a social system is composed [11]. The effective functioning of the family relies on the division of labor among its members, and within the unique ecological context created by the interactions among family members, it directly or indirectly influences the individual’s gender socialization development. Factors such as family members’ biological sex patterns [9], parenting styles of parents [12], parental gender consciousness [13], and parental gender role attitudes [14, 15] all subtly shape an individual’s gender role. In the process of children’s socialization, parents provide gendered everyday items, toys, clothing, or types of games to guide their behaviors and align them with societal expectations for boys or girls, either through explicit demands or implicit expectations [16]. This process is a manifestation of parental gender role attitudes, as children develop their gender roles under the guidance of their parents [15]. Parents transmit their own gender cognitions to their children through the parenting process, resulting in the intergenerational transmission of gender role attitudes [17, 18]. Chinese culture attaches great importance to the inheritance of gender role concepts, such as “男主外, 女主内” (The man goes out to work, while the woman looks after the house), “女做男工, 家道兴隆;男做女工, 越做越穷” (When women do men’s jobs, the family becomes prosperous; when men do women’s jobs, the family becomes poor). These traditional gender role concepts are still reflected in the social gender labor division in contemporary China. The stereotyped cognition of gender roles is passed down from generation to generation through the parenting attitude of the family. Research suggests that the gender attitudes held by grandparents are transmitted indirectly to their grandchildren through their influence of parents on gender cognition [19]. In the Chinese society, grandparents pay great attention to the development of their grandchildren and participate in their daily care [20]. In particular, single-parent families, who lack economic and human resources [21], need more assistance from social networks [22], while grandparents provide the most direct help and support [7]. Therefore, grandparent-parent co-parenting has become an inevitable outcome of the rapid economic development and cultural evolution of most families in China [23]. Grandparents will actively participate in the care of their grandchildren and regard it as their obligation [20], which means that grandparents may directly participate in the upbringing of the children and shape the formation of children’s gender roles [24]. Therefore, parental gender role attitudes, as important factors influencing children’s gender socialization, may undergo intergenerational transmission in both direct and indirect ways.

The family as the structure most able to satisfy the physical and psychological needs of its members and also to maintain the larger society [10]. The absence of one parent in the family will result in an imbalance in family functioning [25]. Research indicates that parental divorce may result in different gender role reinforcement for children of different sexes [26]. Single mothers may tend to emphasize feminine traits such as nurturance, emotional expression, and cooperation, while single fathers may place more emphasis on masculine traits such as independence, competitiveness, and decision-making [27]. This reinforcement may lead to children holding biases towards specific gender characteristics, subsequently influencing their interpersonal interactions [28]. Although some studies have found no significant differences in the outcomes of parenting between same-sex and traditional heterosexual families [29, 30]. However, when there is an unequal division of household labor, it might impact children’s evaluations toward gender groups [31]. Therefore, in single-parent families where one parental gender role is missing, children may lack direct role models in learning about gender roles [32], potentially resulting in an imbalance in the family’s gender ecology. Given the importance of gender balance for family structure and functioning, this study, situated in the context of the increasing prevalence of single-parent families and the growing trend of intergenerational co-parenting between grandparents and parents in China, aims to explore the impact of the intergenerational transmission of gender role attitudes within single-parent families on children’s gender roles.

## Literature review

### The gender roles development of parents and children

Gender roles are a set of behavioral norms corresponding to one’s own gender, acquired through imitation and learning in the process of socialization [33]. The gender role has been categorized into four types: masculine, feminine, undifferentiated, and androgynous [34]. Masculine or feminine roles refer to behaviors that conform to traditional societal norms for men or women. Undifferentiated roles indicate a lack of distinct male or female gender role characteristics, displaying gender ambiguity or overlap. Androgynous roles involve the simultaneous expression of both male and female gender role characteristics, exhibiting clear gender role blending or diversity [34]. Previous studies have shown that androgynous gender roles are the most ideal type, with the best performance in professional achievement [35], school adaptation level [36] and other life aspects. Undifferentiated individuals tend to have higher anxiety levels [37], depression and lower self-assessed level of health [38]. In the family, parents’ gender roles will also affect the construction of children’s gender roles [32]. Children learn male and female gender traits by comparing the differences in behavior directly expressed or unintentionally conveyed by their fathers and mothers [39]. Research has found that changes in single-parent family structure create a freer context for family members to develop gender roles and reduce their traditional gender role formation [40]. Androgynous single fathers have both traditional male traits and easy-going and gentle female traits, while androgynous single mothers are ambitious when they retain female traits [41], and both are more likely to raise androgynous children [42]. On the other hand, single parents often need to play the role of both the father and the mother at the same time. As a result, they are prone to confusion in cognitive and gender role behaviors. It may also make children have unclear gender cognition when observing their parents’ behavior, and are prone to undifferentiated gender roles [43]. Therefore, the question of whether single parents’ gender roles have intergenerational effects on their children’s gender roles remains to be explored.

### The influencing factors of intergenerational transmission of parental child-rearing gender-role attitudes

Parental child-rearing gender-role attitudes refer to the degree which parents show gender role stereotypes when raising their children [14, 44]. The parents’ own gender role values often influence the development of children’s gender roles through their parenting attitudes and behaviors, such as activity arrangements, daily routine, interpersonal relationships, learning focus, housework allocation and emotional expression [15]. In Chinese culture, parents expect boys to have a boys’ “appearance” and girls to have a girls’ “appearance”. “Appearance” is the parent’s expectation or demand that their children exhibit gender behaviors consistent with the parents’ values.

Intergenerational transmission refers to the phenomenon that parents’ abilities, characteristics, behaviors and ideas are transmitted to their children. Empirical research shows that intergenerational transmission is shown through the correlation between parents’ and children’s characteristics, and parents’ characteristics are predictive of their children’s corresponding characteristics [17]. Studies on intergenerational transmission in families are mostly related to parenting attitudes, such as attachment [45], discipline behavior [46], corporal punishment [47], gender roles [32], gender awareness [48], and it was found that there was a correlation in parenting attitudes between the two generations [18]. Individuals generally raise children in the ways that they have observed or experienced, therefore parents who received psychological aggression [49] and physical aggression/corporal punishment [47] in childhood, are more likely to use harsh parenting. Parents who were overprotected in childhood, also adopt the same parenting strategies for their children [50]. As a part of parenting attitudes, parental child-rearing gender-role attitudes may also produce intergenerational transmission.

Although the parents have similar parenting and gender attitudes to the grandparents when they are young [18], there are also maladaptive inconsistent patterns in which parents choose not to repeat the grandparents [51]. In the process of the parents’ upbringing, due to their education and experiences, the books they read, and this era of the gender equality movement, parents’ attitudes may change [52]. Working and living in an increasingly competitive social environment, parents can deeply understand that, in order to raise children with good physical and mental health, and high social adaptability, it is necessary to construct their abilities and values with a more enlightened and equal attitude towards gender roles education [14]. Therefore, parental child-rearing gender-role attitudes of grandparents and parents may not be exactly the same, resulting in different intergenerational transmission types. Thus, this study, when analyzing the intergenerational transmission of parental child-rearing gender-role attitudes, the similarities and differences between grandparents and parents will be further analyzed.

In addition to socio-economic status factors such as the parents’ economic level and education level affecting the intergenerational transmission type, the role of parents’ gender role types and family gender pairs cannot be ignored. Parental child-rearing gender-role attitudes are affected by their own gender role types [14], and the cognition of parents’ gender roles may influence the intergenerational transmission of parental child-rearing gender-role attitudes, which is also a problem worth exploring. Moreover, the difference of gender pairs between parents and children in single-parent families affects the cognition and formation of children’s gender roles [53]. Meanwhile, gender pairs influence the transmission of parental child-rearing gender-role attitudes between two generations. Single parents and children form same-sex dyads and opposite-sex dyads. Taking father-daughter single-parent families as an example of opposite-sex dyads, the parental child-rearing gender-role attitudes received in father’s childhood, may no longer be applicable to cultivate daughters. And the gender difference of parent and child urges parents to adjust the internalized parental child-rearing gender-role attitudes to adapt to the current situation, so the single-parent family gender pair may be an important predictor of the intergenerational transmission type of parental child-rearing gender-role attitudes.

### The influence of intergenerational transmission of parental child-rearing gender-role attitudes on children’s gender role

Gender expectations or requirements in parental child-rearing gender-role attitudes not only come from parents, but also from other family members, which affect the physical and mental development of children [24]. Influenced by traditional cultural concepts, Chinese grandparents generally regard taking care of their children as the responsibility to carry on the family name, and obtain happiness in the dedicated process of taking care of children [54]. In addition, China is in a period of social transition with fierce competition. In order to bring better living conditions to the family, parents devote all their energy to work [21]. Grandparents are also willing to take care of the children for them at home after retirement, and participate in the upbringing of their grandchildren. This makes the grandparent-parent co-parenting system gradually become the main form of current family education. However, in the process of raising their grandchildren, grandparents have the tendency to spoil and overprotective, which will adversely affect the social development of the children [20]. Studies have found that the main gender roles of children being brought up by grandparents are undifferentiated and femininity [24]. This may be because grandparents care too much about grandchildren and do not let them engage in adventurous activities. As a result, male traits such as adventurousness, independence and bravery have not been sufficiently cultivated and exercised, therefore the children tend to be timid and dependent, which is not conducive to their exploration of the external world. It can be seen that the parenting attitudes of grandparents also play an important role in the formation of their offspring’s gender roles.

At the same time, grandparents are an important part of the family constructed by parents, and the parenting styles and constructs of grandparents may either be continued or changed by the parents. Grandparents and parents co-parenting children may cause some differences and disagreements. Studies have shown that differences and conflicts arising from inconsistent parenting beliefs can lead to tension in family relationships and become the cause of children’s problem behaviors [55]. Conversely, if the parenting styles of grandparents and parents are consistent, the children will be less likely to develop problem behaviors [56]. Therefore, it is necessary to pay attention to the property and consistency of parenting attitudes, otherwise it will be difficult to accurately locate their influence on children.

Parents’ gender roles and the intergenerational transmission of parental child-rearing gender-role attitudes may affect children’s gender role development. Other family factors, such as the single-parent family gender pair, family income, parents’ education levels, social-economic status, the number and gender of children affect children’s gender roles, and are also worth exploring. Single-parent families are divided into four family gender pairs by biological sex: father-son families, father-daughter families, mother-son families, and mother-daughter families. Since children construct their own gender mainly by observing and imitating their parents [57], the absent parent in single-parent families makes it easy for teenagers to lose the direct opportunity to understand gender differences and to imitate gender roles, which then affects their gender socialization [8]. Previous studies on the impact of gender pairs in single-parent families on children’s gender roles have shown different results. In father-absent families, girls tend to become relatively more masculine due to mothers’ reliance and pressure, while boys may exhibit fewer masculine due to the lack of fatherly guidance [58]. Other studies have found that boys raised by single mothers have more male traits and show sex-typed gender roles [53]. Moreover, because single parents often have the responsibilities of both the father and mother, their children’s views on gender roles are more flexible and their children are therefore more likely to form androgynous gender roles [59]. Family socioeconomic status is also an important factor in the development of children’s gender roles [15, 60]. Studies have found that individuals with higher income and education levels tend to hold more equal and flexible gender attitudes [60, 61]. Adolescents with siblings have a more solidified concept of different roles in the family [62], meaning that their gender role cognition can be different from only children. Only children are more affected by their parents’ attitudes towards gender than non-only children [62]. Furthermore, children with same-sex siblings tend to make same-sex choices, while those with opposite-sex siblings are likely to make opposite-sex choices [63, 64], indicating that sex differences in the family also interact with the gender identity of the family members. To sum up, various factors in the family will have an impact on the process of individual gender socialization, and thus may affect their social adaptation. Therefore, this study will analyze the intergenerational transmission of parental child-rearing gender-role attitudes in single-parent families and its influence on children’s gender roles, while also bringing various other family factors into consideration for a comprehensive discussion.

### The present study

Previous studies have found that, in the family, parents’ gender roles [32], gender role attitudes of parents, parental child-rearing gender-role attitudes [15], family socioeconomic status [9, 60] and other factors may influence the development of children’s gender roles. It can be seen that parents’ traits and their value systems are one of the main factors in shaping children’s gender roles. From the perspective of cognition, parents will inevitably carry the traces of their children’s grandparents, and therefore influence the gender roles of their children [8], given that grandparents are also an important part of the current education of the family in China [54]. Especially in single-parent families, grandparents can provide strong social support [22]. Therefore, if we ignore the role of grandparents in the gender socialization of single-parent children, we may not be able to fully understand the way family factors shape the gender roles of single parent children. Therefore, this study will start from the intergenerational level to explore the possible impact of the relationship between grandparental and parental child-rearing gender-role attitudes on children in single-parent families. At the same time, in addition to “latent functions” such as parental child-rearing gender-role attitudes, this study also included “explicit functions” such as family socioeconomic status, the number of children in the family, the gender pair of single-parent families, and the gender role of parents in the potential influencing factors. In addition to expanding the understanding of the intergenerational transmission of gender roles in single-parent families, the results of this study can be used as a reference for social workers in designing assistance programs for single-parent families. In conclusion, based on structural function theory, this study comprehensively considers the intergenerational transmission of parental child-rearing gender-role attitudes in single-parent families and the impact of various family factors on children’s gender roles.

Therefore, the research questions of this study are as follows:

1. Are there similarities of gender role types between parents and children in single-parent family?
2. Are there similarities of parental child-rearing gender-role attitudes between grandparents and parents in single-parent family?
3. How do family factors (socio-economic status, family gender pairs, parents’ gender role types) affect intergenerational transmission of parental child-rearing gender-role attitudes in single-parent family?
4. How do family factors (socio-economic status, siblings, family gender pairs, parents’ gender role types, intergenerational transmission of parental child-rearing gender-role attitudes) affect children’s gender role in single-parent family?

## Method

### Participants

With the help of the S Women’s Federation and Education Bureau, this study selected 10 middle schools in the S district, and distributed the scales to single-parent children and their parents from the first grade of junior high school to the third grade of high school. A single-parent family refers to a family in which only one parent lives with the child due to widowhood, divorce, separation, or being unmarried [40]. A total of 1142 child scales and 1126 parent scales were collected. After data sorting, 1126 single-parent family parent-child dyads were obtained. After eliminating those with missing important demographic information, there were 832 matching data remaining. After further eliminating invalid scales, 550 valid matching data were finally obtained, and the scales were matched. The effective response rate of the matched scales was 66.1%. The children of single-parent families ranged in age from 11 to 18 (*M* = 15.04, *SD* = 3.17). 38.4% of the parents’ questionnaire was filled out by fathers (*n* = 211), 61.6% of the parents’ questionnaire was filled out by mothers (*n* = 339); 42.4% of the children’s questionnaire was filled out by boys (*n* = 233), 57.6% of the children’s questionnaire was filled out by girls (*n* = 317). The four types of single-parent families included father-son families (*n* = 87, 15.8%), father-daughter families (*n* = 124, 22.5%), mother-son families (*n* = 146, 26.5%), and mother-daughter families (*n* = 193, 35.1%). The basic information of the participants is shown in Table 1.

**Table 1:** Sample characteristics (*N* = 550)

Variable	***n***	***%***
Parent gender		
Male	211	38.4
Female	339	61.6
Parents’ educational degrees		
Elementary school and below	48	8.7
Junior middle school	245	44.5
High school and technical secondary school	167	30.4
College degree or above	90	16.4
Family income per month		
Less than $687	224	40.7
$687 to $1373	220	40.0
$1374 to $2061	69	12.6
Over $2061	37	6.7
Number of children		
Only one child	331	60.2
Two or more children of the same sex	78	14.2
Two or more children of different sex	141	25.6
Child gender		
Male	233	42.4
Female	317	57.6

### Process

This study was approved by the Ethics Committee of S University, and the purpose and procedures of the study were introduced to the school principal and class teacher before the scales were distributed. With the class teacher’s permission, the information of students from single-parent families in the class was collected. The screening of single-parent families was based on a family in which only the father (or mother) lives with unmarried children under the age of 18 who cannot live independently [65]. Invitations were issued to all parents, along with a study information form and a consent form. The single-parent children and their main caregivers were administered the scales after obtaining the informed consent of the single parents.

In order to protect the personal privacy of participants, all the scales were filled out anonymously. Both scales were assigned numbers by the research team, then two scales were put into two envelopes respectively. The distribution and collection of the scales were completed with the help of the class teacher. The class teacher organized the students to fill out the scales, and then collected them in class. It should be noted that, in order to protect the privacy of the students in the class, other students and parents of the surveyed class participated in filling out the scales.

After the scales were recovered, research team members selected the scales of single-parent families, processed them and eliminated the unmatched data. According to the needs of the research, the deletion of the data in this study mainly follows the following steps: firstly, if the parents or children cannot be matched due to a lack of data, the dyad will be eliminated; secondly, if important demographic information is missing, the corresponding dyad will be excluded; finally, the filling of the items on the scales were checked. If there were unanswered items in the scales, if the data in the scales were answered in a straight-line, or if the answers were regularly meaningless [66], the corresponding dyad was eliminated.

### Measurements

#### Gender-role scale

Liu et al. [33] revised version of Bem’s [67] gender-role inventory was adopted to measure gender roles. The questionnaire comprised of three sub inventories - Masculine scale (16 items), Feminine scale (16 items), and Neutral scale (18 items) - scored using a seven-point Likert-type scale ranging from “completely inconsistent” to “completely consistent”. The neutral scale was not scored, which has an interference effect. The internal consistency coefficients for the original masculine and feminine scale were 0.89 and 0.84 respectively. In this study, the internal consistency coefficient was 0.95. The classification of gender role types was based on androgyny theory [67], using the median method. The mean and median scores of the masculine and feminine sub-scale inventories were calculated separately, and then the mean was compared with the median of each subscale. Individuals whose masculine and feminine mean scores were lower than the corresponding median scores were named undifferentiated, whose male and female positive traits were weak. Individuals whose feminine mean score was higher than the feminine median score were named feminine, meaning their female positive traits are strong. Individuals whose masculine mean score was higher than the masculine median score were named masculine, their male positive traits are strong. Individuals whose masculine and feminine mean scores were higher than the corresponding median scores were named androgynous, with strong male and female positive traits [34]. Further considering the relationship between an individual’s biological sex and their gender roles [68], if an individual’s gender role is consistent with their sex (such as male masculinization and female feminization) is called sex-typed, while male feminization and female masculinization are called reversed [69]. According to the combination of different genders and different gender role types, gender roles can be divided into androgynous, sex-typed, undifferentiated, and reversed. In this study, the median values for parents’ male and female traits were 4.94 and 5.13 respectively, and the median values for children’s male and female traits were 5.50 and 5.06 respectively.

### Parental child-rearing gender-role attitudes scale

Chen et al.’s [15] parental child-rearing gender-role attitude scale was used to measure parental child-rearing gender-role attitudes. This scale is an other-report scale, meaning that children filled it out to measure parents’ PCGA, and parents to measure the grandparents’ PCGA. 39 items cover many aspects of parenting, such as perception and personality expectations, behavior discipline, housework division, leisure activities, material environment, career development, and values transmission. They are divided into a masculinity rearing score (20 items) and a femininity rearing score (19 items). A five-point Likert-type scale was used, ranging from “strongly disagree” to “strongly agree”. After that, the masculinity rearing dimension score and the femininity rearing dimension score were summed up and averaged, respectively, to obtain masculinity rearing score and femininity rearing score. The higher the masculinity rearing score, the more attention parents paid to cultivate children’s male traits; the higher the femininity rearing score, the more attention parents paid to cultivate children’s female traits. The internal consistency coefficient for each dimension of the scale ranged from 0.77 to 0.85.

In order to explore the property of the intergenerational transmission of parental child-rearing gender-role attitudes (ITPCGA), this study divided ITPCGA into different types. The division method of parental child-rearing gender-role attitude types refer to the division of gender role types, and the median division method was used to calculate the average score of the masculinity rearing subscale and femininity rearing subscale, and then the mean score was compared with the median score of each subscale. Individuals whose mean score of masculinity rearing score and femininity rearing score was higher than the respective median scores were named as the enlightened type, indicating that their parents are more open-minded, and at the same time attach importance to the cultivation of positive traits of both males and females. Boys whose masculinity rearing mean score was higher than the median score, while their femininity rearing mean score was lower than the median score, or girls whose femininity rearing mean score was higher than the median score, while their masculinity rearing mean score was lower than the median score were named the traditional type, indicating that their parents follow their children’s biological sex to cultivate them, so that their children meet traditional gender expectations. Boys whose masculinity rearing mean score was lower than the median score, while their femininity rearing mean score was higher than the median score, or girls whose femininity rearing mean score was lower than the median score, while their masculinity rearing mean score was higher than the median score were named the inverted type, indicating that parental rearing was opposite to the children’s biological sex. Individuals whose mean score of masculinity rearing scores and femininity rearing scores was lower than the median score were named the neglect type, indicating that their parents do not pay attention to the cultivation of masculinity and femininity rearing. In this study, the median values for grandparents’ masculinity rearing and femininity rearing were 3.30 and 3.74, while the median values for parents’ masculinity rearing and femininity rearing were 3.30 and 3.68, both respectively.

According to the above classification methods, the parental child-rearing gender-role attitudes of grandparents and parents were divided into four types, and 16 types of intergenerational transmission were also formed (see Table 2). Enlightened and traditional PCGA can be regarded as “normal” PCGA, signifying more positive rearing attitudes, and inverted and neglect PCGA as “deviated” PCGA, that is, relatively negative rearing attitudes [15]. In this study, the intergenerational relationship between “grandparents’ deviated to parents’ normal” and “grandparents’ normal to parents’ normal” PCGA is deemed a benign intergenerational transmission, while that between “grandparents’ normal to parents’ deviated” and “grandparents’ deviation to parents’ deviated” PCGA is deemed an undesirable intergenerational transmission (see Table 2). In single-parent families, the proportions of benign and undesirable ITPCGA were 43.64% and 56.36%, respectively.

**Table 2:** ITPCGA types in single-parent families (*N* = 550)

ITPCGA Types			n (%)
Benign ITPCGA 240 (43.6%)	Normal to Normal 143 (26.0%)	Enlightened to Enlightened	93 (16.9)
		Enlightened to Tradition	18 (3.3)
		Tradition to Tradition	9 (1.6)
		Tradition to Enlightened	23 (4.2)
	Deviated to Normal 97 (17.6%)	Inversion to Enlightened	13 (2.4)
		Inversion to Tradition	9 (1.6)
		Neglect to Enlightened	52 (9.5)
		Neglect to Tradition	23 (4.2)
Undesirable ITPCGA 310 (56.4%)	Normal to Deviated 104 (18.9%)	Enlightened to Inversion	14 (2.5)
		Enlightened to Neglect	45 (8.2)
		Tradition to Inversion	10 (1.8)
		Tradition to Neglect	35 (6.4)
	Deviated to Deviated 206 (37.5%)	Inversion to Inversion	12 (2.2)
		Inversion to Neglect	31 (5.6)
		Neglect to Inversion	27 (4.9)
		Neglect to Neglect	136 (24.7)

**Note:** ITPCGA = intergenerational transmission of parental child-rearing gender-role attitudes

### Demographic variables

In families, demographic variables are important structural and functional variables, which may affect the intergenerational transmission of parental child-rearing gender-role attitudes in single-parent families, and also influence children’s gender roles. Therefore, this study will also collect demographic variables. In the children’s questionnaire, demographic variables included age and gender. In the parents’ questionnaire, demographic variables included gender, age, family type, education level, monthly income, the number of children in the family and their genders. Among them, the socioeconomic status of the family can be calculated by parents’ education level and family income [70].

### Statistical analysis

SPSS 22.0 statistical analysis software was used to collate and analyze the data. Firstly, the chi-squared test was used to analyze the intergenerational relationship between gender roles types and PCGA types. Then, the logistic regression model was used to analyze the factors influencing ITPCGA and children’s gender roles. Because the variables were category variables, multinomial logistic regression was used to fit the model. Multicollinearity tests found that the variance inflation factor (VIF) among the variables was less than 10 (ranging from 1 to 3.5). Tolerance was greater than 0.1 (ranging from 0.28 to 0.86), implying that there was no multicollinearity.

## Results

### Intergenerational similarity in gender roles and parental child-rearing gender-role attitudes in single-parent families

In order to understand the current distribution of gender roles between generations in single-parent families, this study compared the gender role types of parents and children through chi-square analysis (see Table 3). There is no significant difference in the distribution of the four gender role types among parents and children in single-parent families, with both the highest proportion of undifferentiated gender roles. There is consistency in the distribution of parents’ and children’s four gender role types in single-parent families. In order to understand the distribution of parental child-rearing gender-role attitude types between grandparents and parents in single-parent families, this study compared the parental child-rearing gender-role attitude types of grandparents and parents through chi-square analysis. There was no significant difference in the distribution of the four parental child-rearing gender-role attitude types between grandparents and parents in single-parent families, with both the highest proportion of neglect type (see Table 3). There is consistency in the distribution of the grandparents’ and parents’ four parental child-rearing gender-role attitude types in single-parent families.

**Table 3:** Distribution of gender-role types and PCGA types between generations (*N* = 550)

	Gender-role types n (%)						PCGA types n (%)				
	Undifferentiated	Sex-typed	Reversed	Androgynous	*χ²*		Neglect type	Traditional type	Inverted type	Enlightened type	*χ²*
G2	251 (45.6)	62 (11.3)	55 (10.0)	182 (33.1)	1.78	G1	238 (43.3)	77 (14.0)	65 (11.8)	170 (30.9)	2.87
G3	236 (42.9)	58 (10.6)	53 (11.6)	203 (36.9)		G2	247 (44.9)	59 (10.7)	63 (11.5)	181 (32.9)	
*χ²*	0.15	0.15	0.04	1.76			0.02	2.68	0.03	0.45	

**Note:** PCGA = parental child-rearing gender-role attitudes, G1 = Grandparents, G2 = Parents, G3 = Children

### Factors influencing the intergenerational transmission types of parental child-rearing gender-role attitudes in single parent families

This study used binary logistic stepwise regression analysis to analyze the influence of family socio-economic status (SES), single-parent family gender pairs, and parents’ gender roles on ITPCGA types to establish the family factors influencing ITPCGA types and their effects on the ITPCGA types prediction model. The chi-square value of the log-likelihood ratio of the model was 706.57, which is significant at a 1% significance level (*p* < 0.001), indicating an acceptable model fit. The analysis results are shown in Table 4.

**Table 4:** Factors affecting ITPCGA types of single-parent families (*N* = 550)

Predictor	***B***	***SE***	***p***	***OR***
SES	0.08	0.19	0.663	1.09
Father-son family	−0.19	0.28	0.495	0.83
Father-daughter family	−0.58	0.25	0.019	0.56
Mother-son family	−0.26	0.23	0.268	0.78
G2 gender role (Undifferentiated)	−1.15	0.21	0.000	0.32
G2 gender role (Sex-typed)	−1.24	0.32	0.000	0.29
G2 gender role (Reversed)	−0.55	0.32	0.082	0.58

**Note:** ITPCGA = intergenerational transmission of parental child-rearing gender-role attitudes, SES = social economic status, G2 = Parents

Table 4 shows that family SES had no significant influence on ITPCGA types. Compared with mother-daughter families, father-daughter families significant negative predicted benign ITPCGA, with an occurrence ratio (OR) 0.56 times that of mother-daughter families. Parents’ undifferentiated gender roles significant negative predicted benign ITPCGA than parents’ androgynous gender roles, with an OR 0.32 times that of parents’ androgynous gender roles. Parents’ sex-typed gender roles also significant negative predicted benign ITPCGA, with a benign ITPCGA OR 0.29 times that of parents’ androgynous gender roles.

### The influence of family factors on children’s gender roles types in single-parent families

Multinomial logistic regression was used to analyze the impact of family factors on children’s gender roles, including SES (high and low), sibling types (only child, same-sex siblings, opposite-sex siblings), single-parent family gender pairs (father-son, father-daughter, mother-son, mother-daughter), parents’ gender-role types (undifferentiated, sex-typed, reversed, androgynous), and ITPCGA types (benign and undesirable). First, the fit of the whole model was determined. When the independent variable was added, the − 2 times log-likelihood of the model decreased from 496.80 to 369.23 (*p* < 0.001), indicating a good model fit. The multinomial logistic regression results are shown in Table 5.

**Table 5:** Influence of family factors on children’s gender role types in single-parent families (*N* = 550)

	G3 Undifferentiated			G3 Sex-typed			G3 Reversed		
	***B***	***p***	***OR***	***B***	***p***	***OR***	***B***	***p***	***OR***
	−2.39	0.000		−1.96	0.018		−1.74	0.021	
SES	0.28	0.214	1.32	0.12	0.714	1.13	0.12	0.715	1.13
Only one child	0.74	0.218	2.10	−0.05	0.953	0.96	−0.68	0.295	0.51
Two or more children of the same sex	0.33	0.611	1.39	−0.14	0.867	0.87	−1.49	0.074	0.23
Father-son family	0.18	0.569	1.20	−1.51	0.022	0.22	−1.46	0.064	0.23
Father-daughter family	−0.11	0.722	0.90	−0.05	0.887	0.95	−0.59	0.242	0.56
Mother-son family	0.36	0.212	1.43	−1.60	0.006	0.20	0.92	0.014	2.50
G2 Undifferentiated	1.65	0.000	5.20	1.33	0.001	3.79	0.76	0.053	2.15
G2 Sex-typed	0.25	0.533	1.28	1.84	0.000	6.29	0.38	0.487	1.46
G2 Reversed	0.54	0.155	1.72	0.21	0.769	1.23	1.11	0.024	3.05
Undesirable ITPCGA	1.49	0.000	4.44	0.62	0.061	1.85	1.01	0.003	2.74

**Note:** ITPCGA = intergenerational transmission of parental child-rearing gender-role attitudes, SES = social economic status, G2 = Parents, G3 = Children

Table 5 shows that family SES and sibling types did not significantly influence children’s gender roles, while single-parent family gender pairs, parents’ gender-role types and ITPCGA types did. The OR of children’s sex-typed gender roles in father-son families was 0.22 times that in mother-daughter families (comparison between sex-typed and androgynous). The OR of children’s sex-typed gender roles in mother-son families was 0.20 times that in mother-daughter families (comparison between sex-typed and androgynous). The OR of children’s reversed gender roles in mother-son families was 2.50 times that in mother-daughter families (comparison between reversed and androgynous). The OR of children’s sex-typed gender roles in parents’ undifferentiated gender roles was 3.79 times higher than in parents’ androgyny (comparison between sex-typed and androgynous). The OR of children’s sex-typed gender roles in parents’ sex-typed gender roles was 6.29 times higher than in parents’ androgynous gender roles (comparison between sex-typed and androgynous). The OR of children’s reversed gender roles in parents’ reversed gender roles was 3.05 times higher than in parents’ androgynous gender roles (comparison between reversed and androgynous). The OR of children’s undifferentiated gender roles in undesirable ITPCGA was 4.44 times higher than in benign ITPCGA (comparison between undifferentiated and androgynous). The OR of children’s reversed gender roles in undesirable ITPCGA was 2.74 times that in benign ITPCGA (comparison between reversed with androgynous).

## Discussion

Based on structural function theory, this study explored the intergenerational similarity of gender role types and parental child-rearing gender-role attitude types in single-parent families, and comprehensively considered the intergenerational transmission types of parental child-rearing gender-role attitudes in single-parent families and its impact on children’s gender role. The results showed that the distribution of parents’ and children’s gender role types was consistent. There were also similarities in parental child-rearing gender-role attitude types between grandparents and parents. In terms of the intergenerational transmission of parental child-rearing gender-role attitudes and its influencing factors, it was found that the proportion of undesirable intergenerational transmission was higher than that of benign ITPCGA, and that gender pairs and parents’ gender roles affected ITPCGA. Multiple logistic regression results from single-parent families showed that single-parent family gender pairs, parents’ gender roles, and intergenerational transmission of parental child-rearing gender-role attitudes significantly affected children’s gender roles.

### Analysis of intergenerational similarity in gender role types and PCGA types in single-parent families

Difference analysis between parents’ and children’s gender roles showed that the distribution of the four gender role types of parents and children was consistent, with the proportion of undifferentiated gender roles being highest. This is basically consistent with the conclusions of previous studies. Parents’ and children’s gender roles were consistent in distribution, possibly due to the intergenerational transmission of gender roles [32]. Children in single-parent families cannot construct their gender roles by comparing their father’s and mother’s gender role. Therefore, their gender roles are more likely to resemble those of their primary caregiver [41]. In addition, a survey conducted by Jiang et al. [71] found that parents’ and children’s gender role distribution in single-parent families was also highest in undifferentiated gender roles, followed by androgynous, sex-typed, and reversed. This may be because the absence of one parent will lead to children’s lack of observation and imitation of objects of a certain gender, making it difficult for them to understand gender differences and imitate corresponding gendered behaviors and easier to form undifferentiated gender roles [71].

There were similarities between grandparents and parents in terms of PCGA types, with the highest proportion being neglect types. This similarity between the two generations may be because the current generation’s rearing attitudes have implicit and explicit attributes that directly affect the next generation’s attitudes through explicit rearing behaviors and indirectly affect them through subtle influences [72]. Grandparents’ and parents’ PCGAs were mainly neglect type. Grandparents may have ignored parents’ gender education because gender roles were not understood in their times, resulting in a lack of gender education in China. Due to grandparents’ lack of gender equality education, they may not know the importance of gender education. Coupled with the high pressure of life in single-parent families, most single parents are busy with their livelihoods and pay more attention to their children’s academic performances. Under the interlacing of these factors, neglect type PCGA was most common in grandparents and parents.

### The influence of family factors on ITPCGA in single-parent families

Undesirable ITPCGA was more common than benign ITPCGA in single family, meaning the ratio of “deviation to deviation” and “normal to deviation” was much higher. This suggests that the absence of a father/mother from a single-parent family, insufficient economic and social resources, and a lack of gender education knowledge and resources, coupled with a busy work schedule, could result in children’s gender role education being neglected. An empirical study showed that a lack of family structure was not conducive to children’s education [73]. Because the proportion of neglect type PCGA was high in single-parent families, the proportion of undesirable ITPCGA was also high, possibly because parents had a high proportion of neglect and inversion PCGA in their childhood and passed those attitudes along to children. PCGA, like general rearing strategies [50], may be transmitted between generations; thus, single-parent families are more likely to have undesirable ITPCGA.

This study also finds that single-parent family gender pairs and parents’ gender roles affect ITPCGA in single-parent families. Specifically, undesirable ITPCGA is more likely in father-daughter families. Previous studies have pointed out that girls’ personality traits are more influenced by their fathers [74]. Fathers in father-daughter families tend to go to one of two extremes when raising their daughters. They are either overprotective and overindulgent, or extensively educated and overly permissive, making their daughters’ gender roles more likely to deviate. This may be because Chinese fathers are influenced by many factors—such as traditional constructs surrounding the division of labor in the family—and their duties are gradually locked outside the home. In most families, mothers are responsible for raising their children, and paternal rearing gradually retreats into the family. Additionally, gender education starts late in China and has limited popularity. Parents rely more on their own growth experiences and lack the knowledge needed to offer gender education to their opposite-sex children. Therefore, fathers tend to avoid gender education for girls, thereby showing neglect PCGA, resulting in more undesirable ITPCGA. Sex-typed and undifferentiated gender role types in fathers can better predict undesirable ITPCGA, perhaps because parents’ cognition of sex-typed and undifferentiated gender roles is relatively shallow and narrow, whereas grandparents’ PCGA (which parents encountered in childhood) was more often traditional or neglectful. After becoming parents, they passed these two types of PCGA to children, thereby forming an undesirable ITPCGA. Previous studies confirmed that parents with sex-typed gender roles had a more traditional attitude towards gender role education, emphasizing the differences between boys and girls and that their children’s behaviors should conform to their gender-role characteristics [44]. Thus, parents’ sex-typed and undifferentiated gender roles predict undesirable ITPCGA.

### The influence of family factors on children’s gender roles in single-parent families

Multinomial logistic regression showed that single-parent family gender pairs, parents’ gender roles, and ITPCGA types significantly influenced children’s gender roles. The proportion of sons’ sex-typed gender roles in father-son and mother-son families was relatively low, possibly because boys are more susceptible to a lack of family structure and function. Previous studies have pointed out that boys in single-parent families are more affected in psychological and emotional aspects, show more resistance, and display poor socialization in the face of changes in family structure, which may lead to poor male trait scores. Research also pointed out that single fathers spend very little time on childcare activities [75], indicating that the father role is missing in both mother-son and father-son families, which is not conducive to forming boys’ masculinity.

Sons’ gender roles were more often reversed in mother-son families than daughters’ gender roles. This might be because, in single-mother households, sons lack the role modeling and guidance of fathers, leading to relatively lower levels of masculinity [58]. They adopt their mother’s personality traits, exhibiting more feminine characteristics, thus forming an androgynous gender role. Additionally, next to families, an individual’s main point of social contact from infancy to their teens is in schools, which have more female teachers than males; in OECD countries, the proportion of female teachers increased from 61% in 2005 to 68% in 2014 [76]. Boys are effectively surrounded by female teachers in their maturation period, making it easier for them to imitate female gender role objects. Of course, female teachers also provide female role models for sons raised by single fathers, relatively reducing the possibility that these children will have atypical gender roles; however, as sons raised by single mothers largely lack the opportunity to observe and contact male role models, their tendency towards atypical gender roles will be greater [71].

When parents’ gender roles were undifferentiated and sex-typed, children were more likely to adopt sex-typed gender roles; when parents’ gender roles were reversed, children were more likely to be reversed as well. Children’s gender roles were more consistent with parents when the latter had sex-typed and reversed gender roles (i.e., having one gender trait that was much higher than the other [34] and more unitary gender traits. This may attract children’s attention, increasing the likelihood that children will have the same gender roles as their parents. Previous studies have pointed out that parents with sex-typed gender roles have more traditional PCGA [44] and that their children’s gender roles are more masculine or feminine. When parents’ gender roles were undifferentiated, children’s gender roles were more sex-typed. Undifferentiated parents’ masculinity and femininity were lower than the median [34], and their gender traits were weak, making it difficult for children to observe and imitate them when building their gender roles. Moreover, undifferentiated parents tended to have more neglect type PCGA, less often intervening in their children’s gender role development. Children relied on their neurophysiological bias or self-gender preference, leading to undesirable ITPCGA in the form of sex-typed gender roles [77] and predicting a higher OR for undifferentiated and reversed gender role types. This may be because the ITPCGA within the family structure seriously impacts adolescents.

PCGA shows the expectations, attitudes, and standards that parents will encourage when raising their children [78]. Children will form their gender roles based on constant parental feedback through criticism, prevention, or encouragement [8]. Undesirable ITPCGA in single-parent families means grandparents have neglected or made mistakes in raising parents, and that parents will raise children with the same PCGA. Grandparents’ PCGA influences parents’ gender role formations. Meanwhile, parents’ internalized gender neglect and inversed gender cognition demonstrates in the parenting process, which will make children engage in gender role behaviors that deviate from their own sex and form reversed gender roles. Therefore, undesirable ITPCGA can predict the proportion of children with undifferentiated and reversed gender roles in single-parent families.

### Strengths and limitations

This study focuses on single-parent families and considers the family as a unit to understand how gender roles and PCGA were transmitted in different gender pairs within single-parent families. Unlike in previous studies involving single-parent families, the proportion of fathers and mothers in this study was equal, which can better explain the differences between fathers’ and mothers’ PCGA. Researchers have paid insufficient attention to PCGA as an important factor in the effectiveness of family functioning, especially the combined impact of grandparents’ and parents’ PCGA. This study investigated ITPCGA between grandparents and parents and how it influenced children’s gender roles, proving that PCGA was transmitted intergenerationally and influenced children’s gender role development. It has a good reference value to analyze the views of marriage and love of children from single-parent families and cultivate good social adaptation. In this study, the measurement of PCGA was evaluated by the next generation, meaning that children evaluated parents’ PCGA, and parents evaluated grandparents. This evaluation method eliminated the self-report method’s disadvantages, reflecting parents’ PCGA when raising their children more accurately.

However, there are some limitations to this study. Convenience sampling was adopted, with only samples in S, being selected. Although S has a population of more than 10 million, its economic development level and educational level are relatively high in China, which may affect the generalizability of the research results. Future research could involve multi-regional and multi-cultural sampling to obtain more universal results. Additionally, because this was a cross-sectional study, it is impossible to make causal inferences or note effects over time. Parents’ and children’s gender roles and PCGA are dynamic processes. Future research could include a longitudinal follow-up survey to understand the ITPCGA and gender roles. This study explored ITPCGA in single-parent families. However, due to the lack of comparison between single-parent and two-parent families, it is difficult to determine whether its findings apply only to single-parent families or can be extended to two-parent families, and whether there are differences in different family types. Future studies could test for differences by testing parents and children in different family structures (such as same-sex families). Previous studies have found that the number of children and their family birth order will affect children’s gender role development. For example, the number of boys with reversed gender roles was significantly excessive, suggesting boys may associate more with femininity than masculinity, showing gender identity disorder. In this study, the influence of same-sex siblings on the development of individual gender roles was not considered; only sibling relationship type (only child, same-sex siblings, and opposite-sex siblings) was considered. The influence of sibling birth order on adolescent gender role development was not considered, but could be in future research. Finally, for children in single-parent families, in addition to the influence of the main caregivers, external social factors—e.g., other relatives, teachers, classmates, and information on the Internet—may also be involved [52]. This means that even if one parent is absent, children from single-parent families can develop their gender roles through contact with various external sources to find role models to emulate. Therefore, future research could explore the influence of various social factors on adolescents’ gender role development.

### Conclusion and practical implications

The results of this study highlight the effects of single-parent gender pairs and parents’ gender roles on ITPCGA, which influences the development of children’s gender roles. These results provide an empirical basis for understanding how single-parent families influence children’s gender roles and offer corresponding guidance for developing single-parent children’s gender socialization. For example, while paying attention to all single-parent families, the community should give more help and care to mother-son single-parent families. Also, Single parents should recognize the PCGA they have learned from their original families and plays an important role in family socialization. Only then can we provide children with a benign socialization example and a sound gender socialization environment, so as to promote the formation of children’s androgynous gender roles. In order to avoid the occurrence of undesirable IPCGA in the family, parents must enhance their awareness of gender roles and PCGA.

## Data Availability

Due to the ethical obligation to protect the privacy and anonymity of study participants, this data will not be made publicly available, but may be obtained from the corresponding author if reasonably requested.
